# Eric Topol, MD, Discusses Longevity, AI, and Evidence-Based Medicine

**DOI:** 10.20411/pai.v11i1.1028

**Published:** 2026-04-22

**Authors:** Michael M. Lederman, Leonard H. Calabrese

**Affiliations:** 1 Case Western Reserve University, Cleveland, Ohio; 2 Cleveland Clinic Lerner College of Medicine, Cleveland, Ohio

**Keywords:** Artificial Intelligence in Medicine, Evidence-Based Longevity, Preventive Medicine, Scientific Integrity

## Abstract

In this interview, Eric Topol, MD, reflects on a career defined by a sustained commitment to patient-centered medicine. He describes his early training in cardiology, mentorship grounded in a humanistic approach, and how adaptability has shaped his evolving scientific focus. Dr. Topol emphasizes that remaining responsive to emerging evidence is essential for long-term relevance as a physician-scientist. As artificial intelligence shifts research, publishing, and the practice of medicine, he advises early-career physician-scientists to stay curious and prioritize patient care and scientific credibility.

## MICHAEL M. LEDERMAN, MD

Hello, everyone. Welcome to Expert Exchange, a series of interviews with leading research scientists. Expert Exchange is sponsored by *Pathogens and Immunity*. My name is Michael Lederman, and with me is Len Calabrese. We're editors of *Pathogens and Immunity*.

## LEONARD H. CALABRESE, DO

I'm delighted to welcome my former colleague and friend, Eric Topol, who really is one of the most influential physician-scientists of our time. And certainly, one of the most published and cited clinicians in medicine. Eric is a cardiologist and cardiovascular pioneer who has had a major influence in numerous areas, including genomics, digital health, and of course, artificial intelligence (AI). He's currently the founder and director of the Scripps Research Translational Institute and the executive vice president of Scripps Research. Eric is also a best-selling author of titles including “The Patient Will See You Now” and “Deep Medicine,” which were both prescient and got me thinking about the future of digital medicine and AI. He's always held a view that medicine is fundamentally human. His most recent book, “Super Agers,” is my favorite. It's an evidence-based approach to longevity, and it reflects his interest in immunology.

## ERIC TOPOL, MD

Thank you so much, Len. It's really wonderful to be with you. I sure enjoyed our time in Cleveland together.

## MML

Dr. Topol, you are perhaps one of the most widely cited researchers in the known world. Much of your work has been about immunology and infectious diseases. With this breadth of experience, why did you choose cardiology instead of something really interesting, like infectious diseases or rheumatology?

## ET

Great point. I try to change with the times. When I finished training in the 80s, cardiology was the hottest field, with all these new things like angioplasty, clot-dissolving therapies, etc. Over time, I went back to my college roots of genetics. COVID pulled me into infectious disease, which is something that I hadn't paid nearly enough attention to. When I got into “Super Agers,” I realized that the immune system was, if not the singular most important driver, it's right up there in terms of healthy aging and health span. So, I evolve as new evidence emerges, and I try to have that plasticity. It keeps me going.

## LHC

Who, in your early career, inspired you or mentored you in a way to move you along?

## ET

That's an easy one. I went to the University of California, San Francisco, thinking I'd go into diabetes, endocrinology, because my father went blind at age 49 and had all the complications, and I watched how Kanu Chatterjee ran the coronary care unit, and he told me I had to be a cardiologist.

What was really interesting is that he could examine a patient, and he could tell the pressure in each chamber from just touching the patient or listening. But also, when somebody wasn't doing well, he got very emotional. I had not seen any attending get that emotionally connected with patients. And as you pointed out, the humanistic part of medicine, Len, that impacted me. I wanted to emulate him.

## LHC

And, as you mentioned, you were influenced by the pandemic and other factors. I love the concept of reinvention.

## ET

I never thought I would get involved with COVID. But, early on, in February or March of 2020, I was seeing things that frustrated me. Information that was wrong, like that the disease only affected older people. I thought, where's the trusted source? And that was including the CDC and the WHO. So I decided to start poring over the data to see how I could help, because the sources that were supposed to guide us were completely off track. I wound up heavily involved in COVID for 5 years. I learned a lot, and hopefully I shared some worthwhile insights.

## LHC

I think you left a big handprint on this, and it's certainly a testimony to your being willing to look with new eyes.

## MML

You've been thinking a lot and talking a lot about artificial intelligence. What do you think will be the role of artificial intelligence, in terms of the way we publish our work and the way we do our research? Where do you think this is going?

## ET

We're already seeing a pronounced effect on publications with AI-written, AI-generated papers, and AI-generated books. There are AI-generated movies. And then, of course, there's the problem of hybrid work—you don't know what was done by AI, the author, or collaborators. So, this is still in a turbulent time.

Often, when I'm writing, I'll check Gemini 3 or ChatGPT to see if I'm missing anything, because sometimes the results are better than a conventional search, but I think we are all learning and experimenting. It's a difficult problem for editors and publishers on how to rein this in. The most worrisome thing I'm seeing is AI reviews of papers. That really gets me, because it's just like treating a patient, where you don't have that judgment and experience, and you're relying on the pre-training in these mega models to come up with a critique.

Could it be a great way to get reviews? As you know, it's getting harder to get the experts to do the reviews. So, maybe it has a role in the future, but right now, I think, we just don't know.

## MML

What about the idea that you can use AI as a start? You start your search with one of the AI tools, get citations and ideas, but then you confirm that what you're told isn't a hallucination and that it really reflects what's been done.

## ET

So many times I've caught blatant errors. So, it can help, but only with verification.

## LHC

In full disclosure, I've been an advisor to *Open Evidence* since its early days, and my aspiration is to work on fusing the power of generative AI. There's no reason anyone shouldn't have the information anymore. It's instantaneously available. But maintaining our critical appraisal skills and knowing when doing nothing is the right thing to do, there's a certain ineffability of the human component of using these powerful tools that we're struggling with right now.

## MML

Dr. Topol, we put together a journal that we think is different from any other journal that exists. Our goal is to make life better for science and for scientists, and we do this in a couple of ways.

Len and I were fortunate to share an endowment from the charitable Fasenmeyer Foundation, and we used some of these funds to establish *Pathogens and Immunity*. We are free, open-access; we don't charge authors; we have a system that allows submissions to be completed within 5 minutes; we're format-free; and we pay our reviewers a modest honorarium—not enough for them to buy a car, but enough to show that we appreciate their work. We're trying really hard to get our word out and be recognized by the scientific community. Do you have any suggestions for us?

## ET

You're doing it all right—that's fantastic. What's so frustrating is the idea that you would get a bill for your accepted manuscript in a top-tier journal for several thousand dollars. That's grotesque when these journals are making absolutely ridiculous profits. And, it's supposed to be a great honor to be a reviewer, right? But it takes time to do it right, and basically, you're being taken advantage of by these publishing entities that are just reeling in billions of dollars every year. So, you get to resent that.

Your ability to get an endowment is the perfect solution.

## MML

It was. And in fact, if other universities, if other medical schools, want to do the same in their fields, it doesn't take much to fund a journal like ours.

I have another question for you. As I mentioned, you are perhaps one of the most highly cited researchers in the world. Do you have a favorite of the works that you've done that you think is the most important thing that you've contributed to the biomedical literature?

## ET

Thank you. Right now, the thing I think is the most useful is the “Super Agers” book. The reason I say that is that this is where I lay out a new path for medicine. Instead of treat, treat, treat, which is what we do now, it's about prevent, prevent, prevent. And the whole idea is that we have the capacity now to take on the 3 age-related diseases that are compromising our health span: cardiovascular, cancer, and neurodegenerative diseases. We could never do that before. And the ability to use multimodal AI and bring in all these layers of data, including new ones, such as proteomics, biomarkers, genomics, etc. We can't do this as humans, but we can with multimodal AI, so I believe we now have the ability to make predictions—and they're getting more and more impressive—and to prevent. And now our tools for prevention are just getting so much richer, beyond lifestyle factors. So, I'm really excited about the next phase of medicine and changing the world from reactive to preventive. We talked about it forever, but we have never really acted upon it, and now's our chance.

## LHC

Eric, you have been involved in so many areas that have kind of put you in the hot seat in the public eye. I will assert and document that your book is clearly evidence-based, and that's really the strength of your work. But longevity medicine is a field rife with the opposite of what evidence-based medicine stands for. Everyone wants to offer longevity services. How do you recommend crafting evidence-based information? It's not just about having a good doctor who's really focused on a field, but also about not going over the edge.

## ET

You're absolutely right. We have this fact-free world of promoting longevity, and there are physicians and academics involved. They're selling anti-aging supplements or sharing information without any evidence. And some of this is dangerous, no less costly. We have longevity clinics promoting they will manage your longevity for ridiculous amounts of money.

There's another side to this story, which is actually very impressive. There are biotech companies with great science in mice and rats—not in humans —whereby they're reversing aging and have achieved marked slowing of aging through cellular reprogramming, senolytics, and other interesting strategies. Maybe it'll click someday.

So, we have this admixture of these ridiculous anti-aging supplements and salespeople—predators—without data. And then we have some really good science that has to go through additional work and trials to prove itself.

What we have now is what I propose: we can do this now. We might not ever reverse or slow aging. It's kind of inevitable. It's a biological process. But what we can do is prevent the age-related diseases that will occur in each person. And we know this in advance—20 years in advance, which is so big—and that gives us time to work with it. These ideas of reversing aging are very intriguing. I find them scientifically alluring, but what are we going to do right now? And why does the *health span* of the average American end at around age 64 years with a major age-related disease, whereas their *lifespan* is 79 or 80 years? Why can't we do something to make their health span at least as good as their lifespan, if not far better?

## LHC

That's well said, and I have a lot of ongoing concerns about how this is being marketed and absorbed by people, and your voice has been very powerful in this, and I thank you.

## ET

It doesn't get as much popularity as the people and the books that have all the secrets. The problem, as you both know, is that we have a large anti-medical establishment. And so, those who appeal to the anti-establishment get a lot more traction.

## MML

They do indeed.

Well, Dr. Topol, surrounding us all, and throughout our country, our institutions, colleges, and medical schools, are young physician scientists. What kind of advice would you give those focused on a career in biomedical science, a research career as a physician-scientist?

## ET

Staying curious is so fundamental. Never losing touch. The practice of medicine is about your patients. But I also think getting grounded in AI will be important. This will be the most transformative force of our time. So, learn the nuances and understand how far you can take it and its limitations. I think that's going to be important. There's no curriculum to bring AI to the forefront in our medical schools, and there should be.

## MML

When we started our careers, there was this concept of the triple threat. You wanted to be a good physician, a good teacher, and a good laboratory researcher. Must we now be quadruple threats and add to that expertise, interpreting what we learn from artificial intelligence?

## ET

No, I don't think so. I think ultimately, AI will be embedded into the research and in our patient care. Eventually, we won't talk about it anymore, it'll be just part of the augmented human performance. It's going to take a while to get to that point. So, while we're fascinated with it, and also at times very discouraged by it, eventually, a lot of the extraordinary advantages will become a routine part of our practice of medicine. It'll still be the triple threat; we don't need to add another dimension.

## MML

What's the greatest threat to biomedical science, to healthcare, and what is it that gives you hope right now?

## ET

The greatest threat is our current administration. Whether it's being DOGEd, not recognizing the importance of investing in biomedicine and biomedical research, or putting out information that is not evidence-based. We've never had medicine upended like it is today.

What I am optimistic about is that great science is still prevailing. Every day, I try to post on X, or Bluesky, or Substack, or write essays to show that we're not losing momentum. Really great science is continuing, whether that's for autoimmune diseases with T cell engineering or vaccines that help prevent dementia and Alzheimer's. That excites me. We will not cave just because we have an anti-force.

## MML

We will not cave. Dr. Topol, thank you so much for giving us this time. I look forward to reading more from you and your Substack about what we should be thinking about.

## ET

Thank you both, and thanks for the great work that you're doing.

## SUPPLEMENTARY VIDEO

Supplementary videos are available at the *Pathogens and Immunity* YouTube channel.

